# Protective Role of Lycopene in Subjects with Liver Disease: NUTRIHEP Study

**DOI:** 10.3390/nu16040562

**Published:** 2024-02-18

**Authors:** Rossella Donghia, Angelo Campanella, Caterina Bonfiglio, Francesco Cuccaro, Rossella Tatoli, Gianluigi Giannelli

**Affiliations:** 1 National Institute of Gastroenterology—IRCCS “Saverio de Bellis”, 70013 Castellana Grotte, Italy; angelo.campanella@irccsdebellis.it (A.C.); catia.bonfiglio@irccsdebellis.it (C.B.); rossella.tatoli@irccsdebellis.it (R.T.); gianluigi.giannelli@irccsdebellis.it (G.G.); 2 Local Health Unit—Barletta-Andria-Trani, 76121 Barletta, Italy; francescocuccaroepi@gmail.com

**Keywords:** lycopene, liver, antioxidant

## Abstract

Background: Liver diseases are constantly increasing throughout the world and are often associated with other diseases, but above all they are caused by improper diet. Adherence to a diet with abundant vegetables has now been widely demonstrated to be important in combating this pathological condition. The aim of this study was to explore the protective role of lycopene (LYC) extracts from cooked and fresh tomato. Methods: The study cohort included 969 participants assessed in the NUTRIHEP cohort (2005–2006) and the associated follow-up (2014–2016), divided into two groups, based on liver condition: NAFLD, or AFLD and FLD. Results: The results indicated a statistical significance of LYC consumption, showing a protective role against liver disease, the best concentration being 9.50 mg/die, with an RR value of 0.59, *p* = 0.01, 0.39 to 0.90 at 95% C.I., and RRR = 0.40, *p* = 0.002, 0.22 to 0.71 at 95% C.I. Conclusions: The protective role of LYC extracts from tomato has not been amply demonstrated in humans. We conclude that this is one of the few papers in the literature to evaluate the protective effect of LYC against liver disease, as well as how this molecule could be used in future possible treatments. Utilizing lycopene as a supplement alone or in combination with other foods could be useful for developing treatments with reduced contraindications.

## 1. Introduction

Nonalcoholic fatty liver disease (NAFLD) is the most common cause of liver dysfunction worldwide. It affects an increasingly large segment of adults, reaching rates of 25% in Western countries [1], and up to 75% in patients with obesity or type 2 diabetes mellitus [2]. This condition is also increasingly diagnosed in children and teenagers [3]. NAFLD is a liver disorder defined by the accumulation of fat in more than 5% of hepatocytes, in the absence of significant alcohol consumption, drug use, or any other liver disease [4]. It covers a spectrum of hepatic diseases, ranging from simple steatosis to nonalcoholic steatohepatitis (NASH), which can evolve to cirrhosis, liver failure, and hepatocellular carcinoma [5].

Several studies have indicated chronic inflammation and oxidative stress as factors in the initiation and progression of NAFLD [6]. The “multiple hits” theory is the most accepted theory to explain the complex pathogenesis of NAFLD [7]. According to this theory, the close crosstalk between genetics and environmental factors leads to the occurrence of multiple hits, including inflammation, oxidative stress, lipid peroxidation, and mitochondrial dysfunction, promoting the onset of NAFLD and its progression to NASH. 

Two different steps, or “hits”, can be identified: the first is the accumulation of triglycerides in hepatocytes, while the second is oxidative stress [8] that results in lipid peroxidation, mitochondrial dysfunction, and inflammation [7,8,9,10]. 

Currently, no specific drug or therapy is available for the treatment of NAFLD, but international guidelines recommend a lifestyle-based approach, particularly a healthy diet [https://www.iss.it/-/snlg-steatosi-epatica-non-alcolica, accessed on 21 November 2023]. It is now known how dietary factors can modulate hepatic steatosis and counteract liver damage, preventing the evolution of NAFLD into NASH [9]. Some studies have reported the positive effects of the dietary intake of antioxidants from fruits and vegetables on the biomarkers of hepatic damage, in both human and animal models [11,12,13]. 

Recently, among dietary antioxidants, particular attention has been paid to LYC, a phytochemical belonging to the carotenoid family. LYC is abundantly found in red vegetables and fruits, being responsible for their characteristic color: tomatoes, papaya, gac fruit, pink grapefruit, pink guava, carrots, and watermelon [14]. The level of its bioactive properties is influenced by many factors, such as bioavailability, metabolism, isomerization, or interactions with other carotenoids [15]. In nature, LYC occurs in its trans isoform. Thermal treatments, such as cooking, increase the bioavailability of LYC due to its trans-to-cis isomerization [16]. It is assumed that thanks to the cis form, LYC is highly bioavailable in the human diet [17,18]. 

In addition, some studies suggest that an increase in the bioavailability of LYC as a result of its trans-to-cis isomerization is due to the addition of sulfur compounds contained in garlic and onion [19]. Since it is a lipid-soluble substance, consumption together with dietary fat sources amplifies its bioavailability [20]. The main sources of LYC in the diet are tomatoes and tomato-based products [21,22]. In the European population, LYC consumption ranges between 5 and 7 mg/die [23], and more than 80% of its dietary daily intake comes from tomato products [24]. Several studies have demonstrated many significant health benefits of LYC [25]. Due to its structure and lipophilic nature, LYC shows anti-inflammatory and antioxidant effects [26,27]. It is a much more potent antioxidant than either alpha-tocopherol or beta-carotene [28].

The aim of this research is to evaluate the protective role of lycopene derived from the diet in a cohort from Southern Italy, demonstrating on large numbers the effect already discussed in the literature but based on in vitro studies.

## 2. Materials and Methods

### 2.1. Study Population

The NUTRIHEP study is a cohort extracted in 2005–2006 from the medical records of general practitioners in the municipality of Putignano (≥18 years) to minimize errors regarding the distribution of the population sample by age and gender. Furthermore, Italian law requires the presence of a family doctor, allowing the overlap of medical and census data. Participants were interviewed at baseline in 2004–2005 by trained physicians and/or nutritionists to collect information on their sociodemographic characteristics, health status, personal history, and lifestyle factors such as smoking, education level (International Standard Classification of Education), marital status, and eating habits [29]. Weight was measured on an electronic scale (SECA©) and recorded to the nearest 0.1 kg. Height was measured with a wall-mounted stadiometer (SECA©) and recorded to the nearest 1 cm. From 2014 to 2018, all eligible subjects, starting from NUTRIHEP participants, were invited to take part in the follow-up. A total of 1426 subjects responded. The subjects underwent the same protocol as at the first enrollment. 

NAFLD was diagnosed in subjects with hepatic steatosis on ultrasound, without AFL (alcoholic fatty liver; the cutoffs for AFL were 30 g/day for men and 20 g/day for women [30]) or drug-induced fatty liver disease (e.g., corticosteroids, valproic acid, amiodarone, hepatitis C or B viral infections, or other disorders) [31]. Meanwhile, fatty liver disease (FLD) was diagnosed as a common cause of chronic liver disease, encompassing several liver disorders, ranging from simple steatosis to steatohepatitis, liver fibrosis, cirrhosis, and hepatocellular carcinoma [32].

All participants signed informed consent after receiving complete information about their medical data to be studied. Those enrolled at baseline and follow-up were included in this analysis, investigating a total of 969 subjects. The study was approved by the Ethical Committee of the Minister of Health (DDG-CE-502/2005; DDG-CE-792/2014, on 20 May 2005 and 14 February 2014, respectively).

### 2.2. Dietary Assessments

The validated European Prospective Investigation into Cancer and Nutrition (EPIC) food frequency questionnaire was administered during the visit, and each food item (260 food items) was converted into an average daily intake in grams. LYC intake was calculated based on cooked and fresh tomato intake per day. The quantity of lycopene (mg/day) was obtained from the database (https://fdc.nal.usda.gov/ (accessed on 12 April 2019)).

### 2.3. Statistical Analysis

The patients’ characteristics are reported as means and standard deviations (M ± SD) or medians and interquartile ranges for continuous variables, and as frequencies and percentages (%) for categorical variables. 

We estimated a multiple multinomial logistic regression model using the three-category outcome variable (absent, NAFLD, and AFLD and FLD) as the dependent variable and LYC intake (both continuous and categorical) as predictors. The models were also adjusted for some covariates (gender, age, weight, glucose, triglycerides, AST, cholesterol, and HDL), and estimated coefficients were transformed into relative risk ratios (RRRs). To test the null hypothesis of non-association, the two-tailed probability level was set at 0.05. 

The analyses were conducted with StataCorp 2023 Stata Statistical Software: Release 18 (College Station, TX, USA: StataCorp LLC.), while RStudio (“Prairie Trillium” Release) was used for the plots.

## 3. Results

Table 1 shows the epidemiological and clinical characteristics of the NUTRIHEP cohort at first recall and follow-up. 

As shown, only the subjects who returned in the second half were considered, so the numbers are balanced. Furthermore, as described above, the cohort was divided into patients without liver disease, those with NAFLD, and those with AFLD or FLD.

The multinomial logistic regression models are reported in Table 2. 

LYC intake (gr/die) was inserted as an explained variable in the adjusted models, in both continuous and categorical form, to find the best concentration with a protective role. 

**Figure 1:**
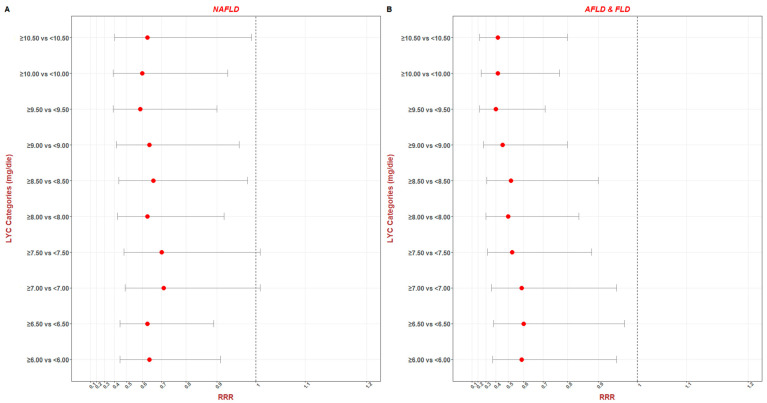
Forest plot of RRR value in the NAFLD (**A**) and AFLD and FLD (**B**) categories.

As a continuous variable, LYC intake showed an RRR < 1 in the NAFLD and the AFLD and FLD groups (RRR = 0.96, *p* = 0.008, 0.93 to 0.99 at 95% C.I., and RRR = 0.94, *p* = 0.006, 0.90 to 0.98 at 95% C.I., respectively). The protective role was better when using LYC as a categorical variable (Figure 1).

The RRR values reached increasingly lower concentrations, the lowest RRR value being at the concentration of 9.50 mg/die, with an RR value of 0.59, *p* = 0.01, 0.39 to 0.90 at 95% C.I., and RRR = 0.40, *p* = 0.002, 0.22 to 0.71 at 95% C.I.

## 4. Discussion

We studied the role of LYC, a phytochemical present in various fruits and vegetables, in modulating the risk of NAFLD, AFLD, and FLD. The innovative aspect of this research lies in its focus on LYC as a potential preventive agent against the progression of liver disease in humans. The study revealed a significant inverse association between LYC intake and the risk of NAFLD, suggesting a potential protective effect. 

Furthermore, this study explored different concentrations of LYC, providing an understanding of its potential dose-dependent impact on NAFLD, AFLD, and FLD. At the same LYC concentrations, the protective effect was more evident against AFLD and FLD than against NAFLD. Previous research associated a high intake of LYC with low waist circumference, as well as with low subcutaneous and visceral fat mass, suggesting that LYC had an impact on adipocyte physiology [33]. 

Moreover, several studies have demonstrated that LYC exerts antioxidant and anti-inflammatory activities, primarily reducing steatosis and obesity-associated disorders in mice [34,35,36]. Other studies have investigated the potential impact of LYC on reducing the risk of cardiovascular disease and suggested mechanisms that involved the regulation of blood pressure and decreased inflammation [37,38]. The antioxidant properties of LYC were studied in relation to cancer prevention. Various studies have provided perspective on the ability of LYC to counteract free radicals, thus reducing the risk of developing various forms of cancer [39]. Other studies have suggested that LYC plays a protective role against ocular disorders and positively modulates the immune response, providing benefits in preventing infections and supporting the immune system [40]. 

Our findings are in line with the current understanding of the pathogenesis of NAFLD, AFLD, and FLD, along with the recognition of their global prevalence and lack of specific therapeutic options. Lifestyle interventions, particularly dietary interventions such as adherence to the Mediterranean diet, have beneficial effects for both the prevention and treatment of NAFLD. Several studies have confirmed the effectiveness of the Mediterranean diet in improving NAFLD, owing to a great variety of specific antioxidants helping to counteract the oxidative stress and inflammation associated with steatosis and, consequently, the extent of liver injury. Several anti-inflammatory and antioxidant mechanisms of LYC have been described that could explain the efficacy of LYC against multifactorial conditions such as NAFLD, AFLD, and FLD. Numerous studies have documented an increase in superoxide dismutase (SOD) and catalase (CAT) activity following LYC administration, highlighting its strong antioxidant potential [41]. 

Additionally, the anti-inflammatory properties of LYC have been corroborated through studies demonstrating a reduction in the expression of different types of cytokines (like TNF-α, IL1, IL6, and IL8), nitric oxide (NO), cyclooxygenase, and chemokines that could modulate the immune system reaction. Furthermore, LYC exhibits a notable impact on lipid metabolism, as evidenced by reductions in serum lipid levels and hepatic cholesterol levels. The precise mechanism underlying these effects is still not fully elucidated, but preclinical studies have striven to unravel the intricacies of LYC’s actions. 

However, there is a notable lack of clinical studies, emphasizing the need for further exploration by the scientific community to comprehensively understand and harness the potential benefits of LYC in the prevention and treatment of NAFLD. LYC’s lipophilic nature plays a crucial role in its anti-inflammatory effects. This property enables it to interact closely with cell membranes, regulating inflammatory mediator signaling pathways and activating antioxidant gene expression. LYC‘s ability to hinder the production of various cytokines, chemokines, nitric oxide, and cyclooxygenase contributes to its modulation of the immune system. Notably, it inhibits the nuclear factor kappa B (NF-κB) signaling pathway, a key player in inflammation, by binding to the inhibitor of NF-κB protein, thus impeding its translocation to the nucleus. 

LYC is found in numerous foods included in the Mediterranean diet, particularly in red vegetables and fruits, such as tomatoes, cherries, pomegranates, pink grapefruit, peppers, carrots, and watermelon. Among these, the most closely studied is the tomato, serving as a vital source of vitamins, minerals, fiber, and carbohydrates, and playing a significant antioxidant role, explained by LYC compounds [14]. Tomato byproducts such as sauce and concentrates are part of the Mediterranean tradition and boast a greater quantity of bioavailable LYC than fresh products. In particular, higher bioavailability is attributed to the impact of food processing, cooking, and isomeric configurations [41]. 

Moreover, the bioavailability of LYC undergoes an increase when it is consumed alongside oils or other fats, such as olive oil. The trans–cis isomerization process of the molecule plays a primary role. In nature, 90% of LYC is found in the trans form, which is the most thermodynamically stable, while in the human body it is mainly present in the cis form, which is the most bioavailable. While the trans LYC form is the primary one in fruits, in cooked and processed products like tomato paste this percentage may decrease to 35%. 

Despite the widespread prevalence of trans isomers, they exhibit lower bioavailability compared to cis isomers. Several studies have indicated that the heightened antioxidant activity of cis isomers may stem from their superior solubility and reduced tendency to self-aggregate in polar media. It has been suggested that the peroxyl radical scavenging capacity of LYC isomers follows the order 5-cis > 9-cis > 13-cis > all-trans, likely owing to the enhanced solubility of cis isomers in comparison to the all-trans form. In contrast, cis isomers of LYC prevail as the typical forms in tissues and plasma. This implies that the majority of ingested trans LYC undergoes conversion to cis isomers through isomerization in the enterocytes, liver, and stomach. Consequently, this process improves the bioavailability and the absorption of LYC [42]. 

Our results showed that the amount of 9.50–10.00 mg/day was the most effective in reducing the risk of steatosis. Although this intake is slightly higher than the actual average intake in our cohort, it is an easily attainable dose based on consumption of LYC-rich foods. For example, to reach this equivalent dose, one would need to consume about four medium-sized fresh tomatoes (calculated as 2.86 mg of LYC per 100 g of tomato; one medium-sized tomato weighs about 125 g) or 50 g of tomato sauce (about 20 mg of LYC per 100 g of tomato sauce) per day [22]. While this study provides valuable insights, it has certain limitations. 

The reliance on self-reported dietary data introduces the possibility of recall bias, potentially affecting the accuracy of LYC intake assessments. The observational nature of the study design limits the establishment of causal relationships. Additionally, the study cohort, extracted from a specific region in Italy, may not fully represent the diversity of global populations, impacting the generalizability of the findings. Furthermore, the potential influence of unmeasured confounding factors on the observed associations cannot be entirely ruled out. 

## 5. Conclusions

In conclusion, our findings suggest that LYC, with its potent anti-inflammatory and antioxidant properties, may serve as a protective factor against the development and progression of steatosis and liver injury. Further clinical studies are needed to confirm these implications. Future studies should aim for more robust methodologies, such as randomized controlled trials, to confirm causation and validate any observed associations. This diet-centric approach could potentially pave the way for personalized nutrition strategies in the management of steatosis. Incorporating LYC-rich foods into dietary recommendations for individuals at risk may become a viable strategy.

## Figures and Tables

**Table 1:** Epidemiological and clinical characteristics of the cohorts, NUTRIHEP 1 and 2.

Parameters *	Cohorts					
	NUTRIHEP 1 (2005–2006) (*n* = 969)			NUTRIHEP 2 (2014–2016) (*n* = 969)		
	Absent (*n* = 748)	NAFLD (*n* = 114)	AFLD and FLD (*n* = 107)	Absent (*n* = 470)	NAFLD (*n* = 368)	AFLD and FLD (*n* = 131)
Age (years)	41.59 ± 13.88	46.67 ± 11.81	54.08 ± 10.23	48.11 ± 13.14	58.41 ± 13.39	61.51 ± 12.41
Age Categories (%)						
<50	424 (56.70)	45 (39.50)	13 (12.10)	283 (60.20)	96 (26.10)	25 (19.10)
≥50	324 (43.30)	69 (60.50)	94 (87.90)	187 (39.80)	272 (73.90)	106 (80.90)
Gender (M) (%)	324 (43.30)	69 (60.50)	94 (87.90)	203 (43.20)	179 (48.60)	105 (80.20)
Smoker (%)						
Never/Former	636 (85.10)	92 (81.40)	87 (81.30)	418 (88.90)	322 (87.70)	110 (84.60)
Current	111 (14.90)	21 (18.60)	20 (18.70)	52 (11.10)	45 (12.30)	20 (15.40)
Marital Status (%)						
Single	208 (28.00)	15 (13.20)	4 (3.70)	91 (19.40)	48 (13.00)	7 (5.40)
Married/Coupled	511 (68.70)	94 (82.50)	99 (92.50)	358 (76.30)	290 (78.80)	113 (86.90)
Separated/Divorced	15 (2.00)	0 (0.00)	1 (0.90)	13 (2.80)	7 (1.90)	4 (3.10)
Widower	10 (1.30)	5 (4.40)	3 (2.80)	7 (1.50)	23 (6.20)	6 (4.60)
Hypertension (Yes) (%)	72 (11.10)	24 (26.40)	29 (29.90)	74 (16.90)	155 (44.50)	55 (44.40)
Diabetes (Yes) (%)	9 (1.20)	4 (3.50)	10 (9.30)	9 (2.10)	34 (9.80)	13 (10.50)
Weight (kg)	66.07 ± 12.19	79.94 ± 17.14	83.93 ± 12.53	66.35 ± 12.06	78.63 ± 14.40	81.72 ± 15.50
BMI (kg/m^2^)	24.43 ± 3.80	29.02 ± 5.64	29.66 ± 3.58	24.88 ± 3.62	30.16 ± 4.81	29.72 ± 4.99
*Blood Parameters*						
Glucose (mmol/L)	5.37 ± 0.87	5.84 ± 0.99	6.13 ± 1.17	4.98 ± 0.54	5.46 ± 1.11	5.79 ± 1.09
Total Cholesterol (mmol/L)	4.88 ± 0.97	5.19 ± 1.02	5.21 ± 0.99	4.85 ± 0.87	5.08 ± 0.95	4.84 ± 0.93
Triglycerides (mmol/L)	1.04 ± 0.69	1.64 ± 0.96	1.68 ± 1.13	0.89 ± 0.69	1.32 ± 0.85	1.41 ± 0.94
HDL (mmol/L)	1.40 ± 0.34	1.20 ± 0.30	1.17 ± 0.23	1.39 ± 0.33	1.27 ± 0.29	1.16 ± 0.28
WBC (K/mcL)	6.13 ± 1.57	6.81 ± 1.64	6.56 ± 1.63	5.63 ± 2.91	6.05 ± 1.62	6.36 ± 1.67
GGT (µkat/L)	0.19 ± 0.15	0.26 ± 0.12	0.27 ± 0.10	0.25 ± 0.14	0.32 ± 0.25	0.38 ± 0.26
AST (µkat/L)	0.18 ± 0.10	0.21 ± 0.08	0.21 ± 0.07	0.35 ± 0.12	0.38 ± 0.30	0.40 ± 0.10
ALT (µkat/L)	0.23 ± 0.21	0.35 ± 0.17	0.33 ± 0.16	0.33 ± 0.15	0.42 ± 0.44	0.43 ± 0.17
Blood Lipids (Yes) (%)	30 (4.60)	4 (4.40)	10 (10.30)	54 (12.30)	61 (17.60)	25 (20.20)
rMed ^^^ (Yes) (%)	8 (6–11)	8 (5–11)	8 (6–11)	9 (7–11)	9 (7–11)	8 (6–10)
Kcal (die)	2106.53 ± 770.46	2064.69 ± 838.32	2157.11 ± 761.54	2075.02 ± 707.08	1993.26 ± 775.25	2199.42 ± 775.94
Cooked Tomato (gr/die)	21.71 ± 24.84	22.10 ± 21.32	26.89 ± 24.09	11.98 ± 14.04	11.28 ± 14.03	16.14 ± 18.53
Fresh Tomato (gr/die)	45.36 ± 43.34	47.30 ± 66.78	51.23 ± 47.74	46.06 ± 46.66	49.27 ± 46.79	42.96 ± 34.48
LYC Intake (mg/die)	6.32 ± 5.43	6.78 ± 5.29	6.79 ± 5.31	4.82 ± 3.76	4.80 ± 3.74	5.48 ± 4.52

* As means and standard deviations (M ± SD) for continuous variables, and as frequencies and percentages (%) for categorical variables; ^^^ as medians and interquartile ranges. Abbreviations: NAFLD, nonalcoholic fatty liver disease; AFLD, alcoholic fatty liver disease; FLD, fatty liver disease; BMI, body mass index; HDL, high-density lipoprotein; WBC, white blood cells; GGT, gamma-glutamyl transferase; AST, aspartate aminotransferase; ALT, alanine transaminase; rMED, relative Mediterranean diet score; LYC, lycopene.

**Table 2:** Multinomial logistic regression analysis of liver condition categories for NUTRIHEP 2 on LYC intake at NUTRIHEP 1 as continuous and categorical variables inserted in the model ^^^.

Parameters	NAFLD ^¥^				AFLD and FLD ^¥^			
	RRR	se (RRR)	*p*	95% C.I.	RRR	se (RRR)	*p*	95% C.I.
LYC *(Continuous)*	0.96	0.02	0.008	0.93 to 0.99	0.94	0.02	0.006	0.90 to 0.98
LYC *(Categorical)*								
≥6.00 vs. <6.00	0.64	0.11	0.12	0.45 to 0.91	0.59	0.14	0.03	0.37 to 0.95
≥6.50 vs. <6.50	0.63	0.11	0.01	0.45 to 0.89	0.60	0.14	0.04	0.38 to 0.97
≥7.00 vs. <7.00	0.71	0.13	0.06	0.49 to 1.01	0.59	0.14	0.03	0.36 to 0.95
≥7.50 vs. <7.50	0.70	0.13	0.06	0.48 to 1.01	0.53	0.14	0.01	0.32 to 0.88
≥8.00 vs. <8.00	0.63	0.12	0.02	0.43 to 0.92	0.50	0.13	0.009	0.30 to 0.84
≥8.50 vs. <8.50	0.66	0.13	0.04	0.44 to 0.98	0.52	0.14	0.02	0.31 to 0.90
≥9.00 vs. <9.00	0.64	0.13	0.03	0.42 to 0.96	0.46	0.13	0.006	0.27 to 0.80
≥9.50 vs. <9.50	0.59	0.13	0.01	0.39 to 0.90	0.40	0.12	0.002	0.22 to 0.71
≥10.00 vs. <10.00	0.60	0.13	0.02	0.39 to 0.93	0.42	0.13	0.004	0.24 to 0.77
≥10.50 vs. <10.50	0.63	0.15	0.05	0.40 to 0.99	0.42	0.14	0.008	0.22 to 0.80

Abbreviations: NAFLD, nonalcoholic fatty liver disease; RRR, relative risk ratio; se(RRR), standard error of RRR; 95% C.I., 95% confidence interval. ^¥^ Reference category: absent. ^ Models adjusted for gender, age, weight, glucose, triglycerides, AST, cholesterol, and HDL in NUTRIHEP 2.

## Data Availability

The original contributions presented in this study are included in the article. Further inquiries can be directed to the corresponding author.
